# Development and Validation of a TLC-Densitometry Method for Histamine Monitoring in Fish and Fishery Products

**DOI:** 10.3390/molecules25163611

**Published:** 2020-08-08

**Authors:** Ayoub Kounnoun, Adnane Louajri, Francesco Cacciola, Hafssa El Cadi, Hajar Bougtaib, Naoual Alahlah, Aicha El Baaboua, Mohamed El Maadoudi

**Affiliations:** 1 Laboratory of Applied Biology and Pathology, Department of Biology, Faculty of Sciences of Tetouan, Abd Al Malek Essaadi University, Tetouan 93000, Morocco; alouajri@hotmail.com (A.L.); hajarbougtaib@gmail.com (H.B.); elbaabou.aicha@gmail.com (A.E.B.); 2 Department of Biomedical, Dental, Morphological and Functional Imaging Sciences, University of Messina, 98125 Messina, Italy; 3 Laboratory of Valorization of Resources and Chemical Engineering, Department of Chemistry, Abdelmalek Essaâdi University, Tangier 90000, Morocco; hafssa.elcadi@yahoo.fr; 4 Regional Analysis and Research Laboratory, National Office of Food Safety ONSSA, Tangier 90000, Morocco; naoual.alahlah@yahoo.fr (N.A.); m.elmaadoudi@gmail.com (M.E.M.)

**Keywords:** histamine, fish, TLC, densitometry, optimization, validation

## Abstract

Histamine poisoning is a significant public health problem. Therefore, the monitoring of histamine content in fish and fishery products is considered to be a crucial measure in the seafood industry. In the present study, a simple and rapid densitometric thin-layer chromatographic (TLC) method for histamine determination in fish samples was developed and validated. The samples were homogenized with 10% trichloroacetic acid and histamine was efficiently extracted. Then, an appropriate derivatization procedure was adopted with dansyl chloride. Once the derivatization was carried out, the samples were applied to silica gel TLC plates and developed by ascending chromatography with chloroform-triethylamine (6:4, *v*/*v*) as the mobile phase. The intensity of the histamine-dansyl derivative spots was measured by densitometry at 365 nm, and the quantitation was performed by BIO-1D image processing software. The validation of this method revealed good linearity and specificity over a concentration range from 6.25 to 100 mg/kg. Adequate precision was shown by relative standard deviations (RSD) smaller than 4.82%, accuracy ranged from −6.88% to 5.28%, and satisfactory recoveries ranging from 93% to 105% were obtained. The Limit of Detection and the Limit of Quantification were calculated at 4.4 mg/kg and 10.5 mg/kg, respectively. In addition, the effectiveness of the proposed method was assessed by the analysis of various samples, and the obtained results were confirmed with those achieved by the HPLC-UV method. Moreover, the developed method was found to be simple, cheap, and suitable for application to analyze several samples simultaneously.

## 1. Introduction

Fish is considered an excellent low-fat source of protein, and the consumption of fish provides many benefits to human health, such as protection against heart diseases and certain cancers [1,2]. However, these beneficial effects might be affected by fish decomposition and the accumulation of chemical contaminants such as biogenic amines [2]. During the decomposition process, the content of the amino acid histidine in fish muscle is converted into histamine, due to the microbial action of the histidine decarboxylase enzyme [3,4,5]. Histamine poisoning, also known as scombroid poisoning, is one of the most common forms of toxicity caused by the consumption of fish worldwide; commonly it is a mild illness, but symptoms can vary from moderate to severe to life-threatening [5,6,7].

Histamine content in fish increases with the process of fish spoilage. Therefore, histamine content has been proposed as a form of chemical evidence of spoilage and freshness. As a consequence, countries such as the USA, through the Food and Drug Administration (FDA), and those of the European Union, by the Commission Regulation (EC) No. 2073/2005, have established regulatory limits for histamine levels in edible fish [8,9,10]. According to the FDA, a concentration of 50 mg/kg is an index of sample deterioration; thus, samples returning results at or above this level cannot be intended for sale or consumption. The European Commission regulation requires that from each sample, nine units must be analyzed, and has set the maximum average level of histamine at 100 mg/kg for fishery products from fish species associated with a high amount of histidine, and 200 mg/kg for fishery products which have undergone enzyme maturation treatment in brine, such as salt-ripened anchovy [11,12,13].

A variety of methods are available for histamine analysis in fish and fishery products, such as enzyme-linked immunosorbent assay (ELISA), colorimetric, and chromatographic methods [14,15,16]. Among these methods, HPLC coupled with different types of detectors have been the most widely used. The HPLC methods were found to offer remarkable specificity and sensitivity, but demand sophisticated and expensive instrumentation, trained operators, and entail high operational costs and time requirements [17,18,19]. For this reason, during official controls and routine analysis, the Association of Official Agricultural Chemists (AOAC) fluorescence method was applied for histamine determination [20], while the HPLC method was used as a confirmation method when a sample tested did not conform to the fluorescence method. The AOAC method is considered sensitive and reproducible; nonetheless, it is complex, time-consuming, and requires laborious purification [17]. As such, increasing attention has been paid to thin-layer chromatography (TLC) as a powerful and simple analysis method. TLC does not require costly equipment and can be implemented for the simultaneous analysis of several samples, which can be very useful for histamine determination in fish samples, so that all nine units that compose a sample can be analyzed on a single plate [19,21,22,23]. In addition, and due to the advancements in the systems for image capture and analysis, TLC can be applied as a quantitative method with significant results [24]. Biogenic amines are highly polar compounds; therefore, most chromatographic methods that have been proposed for its determination in fish and fishery products are based on its derivatization with a specific reagent, such as dansyl chloride or *O*-phthalaldehyde (OPA), to produce fluorescence and strong derivatives [25], which acquire properties that facilitate chromatographic separation such that histamine can be easily detected and quantified [26].

The objective of this study was to establish a facile, rapid, and cost-effective TLC approach for histamine monitoring in fish and fishery products. To this aim, histamine was extracted with trichloroacetic acid, and an effective derivatization method with dansyl chloride was adopted after several optimization tests. Then, one-dimensional thin-layer chromatography was applied for the separation of histamine-dansyl derivatives. Finally, an easy and rapid semi-quantification technique was performed by an image analysis system, where the histamine-dansyl derivatives bands were visualized and quantified. To ensure the application and the effectiveness of the developed method, a validation procedure was carried out according to the following parameters: linearity, specificity, Limit of Detection (LOD) and Limit of Quantification (LOQ), precision, accuracy, and recovery.

## 2. Results and Discussion

### 2.1. Method Development

#### 2.1.1. Derivatization Procedure

The derivatization methods of histamine and other biogenic amines with dansyl chloride have been proposed in previous studies. Among these methods, a dansylation method was tested [18], where 1 mL of histamine standard solution was mixed with 1 mL of borate buffer (0.2 M, pH = 9.2), following which the pH medium was adjusted to 10.0 with sodium hydroxide and 2 mL of dansyl chloride were added. After 1 h of incubation at 55 °C in the dark, 40 µL of the derivatization solution were deposited on the TLC plate, and a mobile phase composed of chloroform-triethylamine (6:4, *v*:*v*) was used for the chromatographic separation. The results showed a good separation of histamine bands on the TLC plate. However, the main drawback of this method is the high limit of detection for consistency. It was reported that the accuracy of the TLC method for histamine determination in fish samples is limited near 50 mg/kg [27].

In certain studies, the saturated sodium bicarbonate solution was used as a derivatization buffer for the dansylation of biogenic amines [28,29]. Thus, the borate buffer was replaced by a saturated NaHCO_3_ solution. After derivatization, with dansyl chloride prepared in acetone, organic and aqueous phases were obtained. However, it is difficult to achieve a good separation when using saturated buffer; consequently, the spotting of the sample from the organic phase on the TLC plate was not easy. Therefore, the toluene solution was used to achieve better separation, and the spotting procedure was rapid and much simpler.

#### 2.1.2. Optimization of Derivatization Procedure

The derivatization process is considered essential, and for successful analysis, it is vital to apply an adequate procedure. To this aim, different volumes of histamine solution, sodium bicarbonate buffer, and toluene solution were tested to find the optimal ratios. The optimal results were obtained when 300 µL of saturated buffer were added to 200 µL of histamine standard solution or sample extract solution, and it was concluded that decreasing or increasing the buffer volume significantly influenced the derivatization between histamine and dansyl chloride. As for toluene solution, a volume of 250 µL was sufficient to show a good separation between the aqueous and organic phases and to concentrate the dansyl amine derivatives in the organic phase.

**Figure 1:**
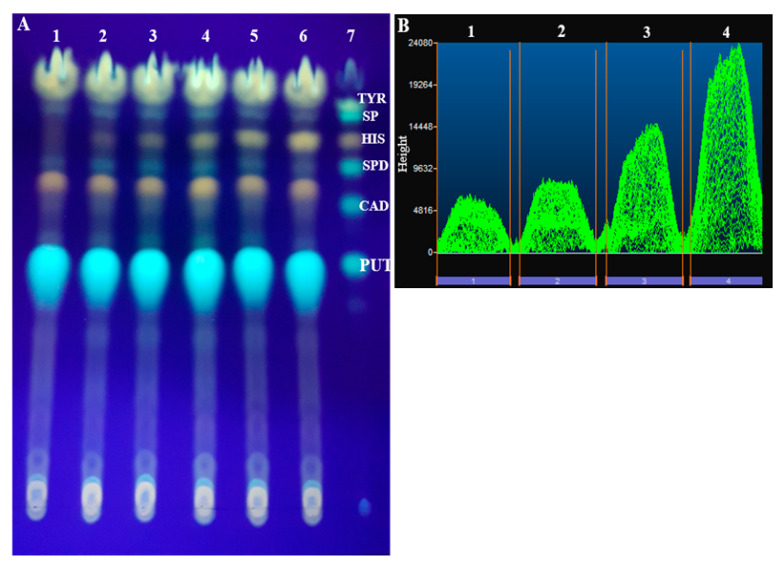
(**A**) Thin-layer chromatographic (TLC) plate of spiked canned tuna visualized under UV lamp at 365 nm: 1 = blank; 2 = 6.25 mg/kg; 3 = 12.5 mg/kg; 4 = 25 mg/kg; 5 = 50 mg/kg; 6 = 100 mg/kg; 7 = Biogenic amine standards; PUT = Putrescine; CAD = Cadaverine; SPD = Spermidine; HIS = Histamine; SP = Spermine; TYR = Tyramine. (**B**) Chromatogram of histamine spots shown with BIO-1D software after visualization of the TLC plate by a Quantum ST5 system: 1 = 6.25 mg/kg; 2 = 12.5 mg/kg; 3 = 25 mg/kg; 4 = 50 mg/kg.

The most common reagent used for the determination of biogenic amines is dansyl chloride [30], and according to the reaction mechanism, its concentration is considered a crucial parameter. In the next set of experiments, the derivatization was performed with three different concentrations of dansyl chloride—0.4%, 0.8%, and 1.5%—in acetone. The quantification of histamine intensity bands was significantly improved by increasing the dansyl chloride concentration from 0.4% to 0.8%. However, increasing the concentration from 0.8% to 1.5% did not show noteworthy variation. After various optimization tests, a successful separation and quantification of histamine was carried out (Figure 1).

#### 2.1.3. Solvent System Effect

Dansyl chloride is a non-specific reagent and was found to react with all the amino compounds including biogenic amines, free amino acids, and ammonia [18]. After the separation of the dansyl derivatives on the TLC plate, different fluorescence spots would be detected. In order to perform a successful quantification of histamine on the TLC plate using an image processing software, several solvent systems were examined. Two solvent systems, namely chloroform:triethylamine (6:4, *v*/*v*), and chloroform:diethyl ether:triethylamine (6:4:1, *v*/*v*/*v*), were evaluated by one-dimensional TLC. Two-dimensional TLC was performed with chloroform:diethyl ether:triethylamine (6:4:1, *v*/*v*/*v*), followed by a chloroform:triethylamine (8:2, *v*/*v*) solvent system.

The results indicate that histamine-dansyl bands presented with a small Rf value and were not well-separated from spermidine derivative spots, when the solvent system chloroform:diethyl ether:triethylamine (6:4:1, *v/v/v*) was used. For the two-dimensional TLC separation, histamine-dansyl spots were developed with a high Rf value, and close to the non-reacting dansyl chloride, with a long developing time. Among the tested solvent systems, the most effective system was chloroform:triethylamine (6:4, *v*/*v*), which showed well-separated, strong, and very clear histamine-dansyl spots, with an Rf value equal to 0.77 (Table 1).

### 2.2. Method Validation

#### 2.2.1. Linearity

To determine the linearity of the proposed method, standard curves were prepared with five points at the following concentrations: 6.25, 12.5, 25, 50, and 100 mg/kg. Each concentration was applied onto a TLC plate with three replications per day over six successive days. The fluorescence histamine spots were analyzed by image processing software after one-dimensional TLC separation, and the calibration curves were constructed by plotting the peak area of the spot density versus the concentration. Twelve calibration curves with linear regression were evaluated in the solvent (n = 6) and by spiking canned tuna extract samples (n = 6). A good linear correlation was revealed between the mean peak area and the amount of histamine, where the calculated determination coefficient (R_2_) ranged from 0.9892 to 0.9997 and a typical standard curve was y = 9,890,660X + 626,037 (R_2_ = 0.999712).

#### 2.2.2. Matrix Effect

**Figure 2:**
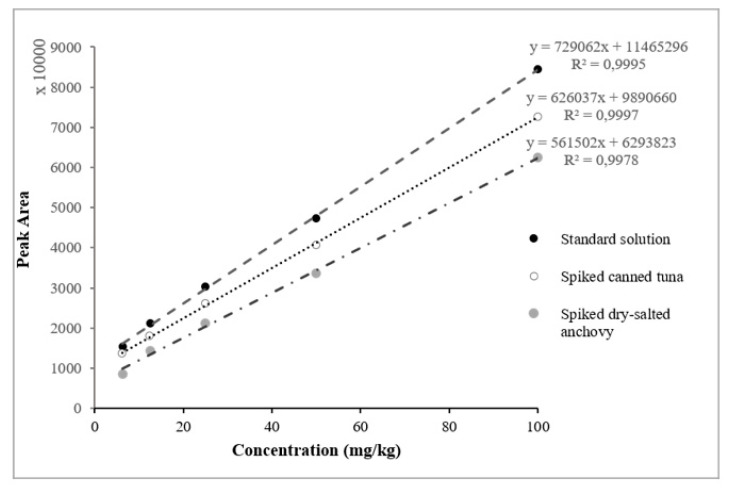
Calibration curves of histamine in solution, in canned tuna, and in dry-salted anchovy.

The application of the calibration curve in the fish matrix is recommended for histamine quantification, due to interference from the matrix effect. To investigate this effect, calibration curves prepared with the solvent were compared with those involving the canned tuna matrix. A significant variation was observed between these curves, and the matrix effect was confirmed, as can be seen in Figure 2. To evaluate the application of the same calibration curve, different calibration curves prepared in various samples, such as fresh sardine, fresh anchovy, fresh mackerel, marinated anchovy, dry salted anchovy, and canned sardine, were compared with the canned tuna validation curves. For fresh, marinated, and canned samples, a non-significant difference was observed, thus the canned tuna curve could be used to analyze these samples. For dry-salted samples, a noteworthy variation was observed (Figure 2), which indicates that the histamine analysis in these samples should be carried out with a specific calibration curve prepared in these matrices. This difference can be explained by the effects of the salt and the enzyme maturation treatment. The matrix effect has been confirmed and reported in previous studies for histamine determination in fish and fishery products [31,32].

#### 2.2.3. Identity and Specificity

To confirm the identity of histamine, spiked samples and standard solutions were deposited on the same TLC plate, and the Rf value of histamine bands was evaluated. To evaluate the specificity of the method, n = 20 blank canned tuna samples and spiked samples with standard solution mixtures of histamine, cadaverine, spermidine, tyramine, spermine, and putrescine were analyzed. In all cases, histamine was clearly separated from other amines and no significant interference was detected at its Rf value.

#### 2.2.4. Limit of Detection (LOD) and Limit of Quantification (LOQ)

LOD and LOQ are calculated by taking the mean sample blank value and adding 3 and 10 times the SD (standard deviation) of repeated measurements, respectively [33]. Canned tuna samples were analyzed with repeated measurements of n = 20, and each analysis was expressed as the analyte concentration. The LOD, determined as the lowest concentration of the histamine that can be detected at a specified level of confidence, was 4.4 mg/kg, and the LOQ, calculated as the lowest concentration of histamine that can be determined with an acceptable level of repeatability, precision, and trueness, was 10.5 mg/kg.

#### 2.2.5. Precision, Accuracy, and Recovery

To examine the precision in terms of repeatability, accuracy (trueness, %), and recovery of the developed method, canned tuna samples were spiked at five different concentrations of histamine (6.25, 12.5, 25, 50, and 100 mg/kg), and were analyzed over six successive days with three replications per day. The precision of the assay was determined by calculating the relative standard deviation (RSD, %) for the repeated measurements, and the accuracy of the method (trueness, %) was evaluated by assessing the agreement between the measured and nominal concentrations of analyzed samples [9,10].

The results are shown in Table 2: the RSD % values of the intra-day repeatability were in the range of 2.19–4.82%, the trueness values for the accuracy ranged around −6.88–5.28%, and the recoveries ranged from 93% to 105%. These values are very satisfactory, and thus the validated TLC method can be considered precise, accurate, and repeatable. Further, the obtained results are in agreement with other data available in the scientific literature about the determination of histamine in fish and fishery products [19,21,34].

### 2.3. Determination of Histamine in Fish and Fishery Products

To demonstrate the effectiveness and to confirm the application of the developed and validated TLC method for the determination of histamine in fish samples, different fish and fishery products were analyzed and the results were compared with those obtained using the international standard HPLC-UV method. The results are given in Table 3 and indicate that the TLC analytical method is accurate and reliable.

## 3. Materials and Methods

### 3.1. Reagents and Chemicals

Biogenic amine standards, histamine, cadaverine, spermidine, spermine, putrescine, and tyramine were obtained from Sigma-Aldrich (St. Louis, MO, USA). Chemical and analytical reagents were obtained as follows: hydrochloric acid, dansyl chloride, and acetone from Sigma (St. Louis, MO, USA); potassium chloride from Panreac (Barcelona, Spain); trichloroacetic acid, sodium hydroxide, boric acid, sodium carbonate, and toluene from Loba Chimie (Mumbai, India). Chloroform and diethyl ether solvents used for TLC separation were purchased from Sigma-Aldrich (St. Louis, MO, USA), and triethylamine from Loba Chimie (Mumbai, India).

### 3.2. Samples

Fresh mackerel fish samples collected for official controls were used for the development of the TLC analytical method, while canned tuna samples were used for the method validation procedure. These samples were homogenized using a hand blender and stored in sterile polyethylene bags at −20 °C. Fresh fish and fishery products, such as fresh sardine, fresh anchovy, canned mackerel, marinated anchovy, dry salted anchovy, and canned sardine were also used.

### 3.3. Determination of Histamine by TLC-Densitometry

The chromatographic separation was performed on TLC aluminium sheets measuring L × W 20 × 20 cm with silica gel 60 F_254_ (Merck KGaA, Darmstadt, Germany). After the derivatization of histamine with dansyl chloride, 50 µL of the sample were spotted at 1.5 cm from the bottom of the plate under dryer flow. The plate development was carried out in darkness, at room temperature, and in a glass chromatographic chamber, with chloroform-triethylamine (6:4, *v*/*v*) solvent system as the mobile phase. The TLC plate was developed up to a distance of 18.5 cm from the bottom, and then was dried in a fume hood at room temperature.

The plate was visualized under an ultraviolet lamp at 365 nm wavelength, then histamine-dansyl derivatives bands were marked, as well as other biogenic amine bands, and the Rf values were determined. For quantitative analysis, the plate was divided into two parts, and each part was detected at 365 nm by a Quantum ST5 1326 series system (Vilber-Lourmat, Marne-la-Vallée, France) equipped with a CCD camera and Vision-Capt software. The semi-quantification was performed using BIO-1D image processing software.

### 3.4. Preparation of Standard Solutions

A histamine standard stock solution of 1000 µg/mL was prepared in 0.1 N HCl. The calibration curve was prepared, by appropriate dilution of histamine stock solution in solvent and in the matrix samples, to obtain aliquots of the following concentrations: 3.75, 6.25, 12.5, 25, and 50 µg/mL, which corresponded to 6.25, 12.5, 25, 50, and 100 mg/kg in fish samples. Other standard solutions of cadaverine, spermidine, spermine, putrescine, and tyramine were prepared separately in 0.1 N HCl at a concentration of 1000 µg/mL. Dansyl chloride solution was prepared by dissolving 0.8 g of dansyl chloride in 100 mL of acetone, which was then stored at −20 °C in a brown glass bottle.

### 3.5. Sample Preparation

#### 3.5.1. Extraction

In brief, 4g of a well-mixed sample were weighed into a 50 mL polypropylene tube. The sample was homogenized with 10 mL of 10% trichloroacetic acid, and the tube was centrifuged for 10 min at 3000 g (C-28 model BOECO centrifuge, Germany). After centrifugation, the supernatant was filtered through a 150 mm membrane filter.

#### 3.5.2. Derivatization

In brief, 200 µL of the filtrate were transferred into a 2 mL centrifuge tube, then 300 µL sodium carbonate solution (6.4 g of Na_2_CO_3_ in 20 mL of distilled water) and 500 µL of dansyl chloride solution were added. The tube was vortexed and incubated for 1 h in the dark at 60 °C. Then, 250 µL of toluene were added to improve the separation between aqueous and organic phases. Finally, 50 µL from the organic phase were deposited on the TLC plate, under a stream of warm air blown out gently from a hair dryer.

### 3.6. Method Validation and Statistical Analysis

Validation and statistical analysis of the developed TLC method was made depending on the following parameters: linearity, specificity, Limit of Detection (LOD) and Limit of Quantification (LOQ), precision, accuracy, and recovery, and carried out according to the European Commission Decision 2002/657/CE, concerning the performance of analytical methods and the interpretation of results and the Afnor standard NF-V03-110 [35,36].

### 3.7. HPLC-UV Method

The international standard HPLC-UV method was used for the analysis of different fish samples [37]. This method involves the use of dansyl chloride as pre-column derivatization reagent, a liquid-liquid extraction method as the purification procedure, and a reverse-phase HPLC separation method, which was carried out by a PerkinElmer Series 200 HPLC system equipped with an autosampler, Kromasil 100-5-C18 column (4.6 × 250 mm, 5 µm), and UV detector.

## 4. Conclusions

A semi-quantification TLC method was developed and validated for the analysis of histamine in fish and fishery products. Following the extraction of histamine, as well as other biogenic amines, and after the optimization of different parameters, a simple derivatization procedure was performed. Then, the biogenic amine-dansyl derivatives were separated on a TLC plate, where the corresponding histamine spots were revealed as strong and very clear spots. Thus, effective identification of histamine was performed by the image capture system, and a successful quantification was carried out. In addition, a comparison was made between the optimized TLC-densitometry method and HPLC method by the determination of histamine in various samples. The developed method has proven to be efficient, inexpensive, and rapid. Thereby, it can be used to concurrently analyze all nine units that compose a sample, according to the European Commission Regulation. Furthermore, and taking into account the validation data, the proposed TLC-densitometry method in this study is suitable to be employed in most laboratories because of its simplicity, rapidity, and efficiency.

## Figures and Tables

**Table 1:** Rf values of the different biogenic amine-dansyl derivatives separated by the TLC method.

Biogenic Amines	TLC Rf Values
**Putrescine**	0.50
**Cadaverine**	0.61
**Spermidine**	0.69
**Histamine**	0.77
**Spermine**	0.82
**Tyramine**	0.85

**Table 2:** Precision, accuracy, and recovery data for histamine in spiked canned tuna samples.

Nominal Concentration (mg/kg)	Observed Concentration ^a^ (Mean mg/kg ± SD)	Intra-Day Precision ^b^ (%)	Inter-Day Precision (%)	Trueness ^c^ (%)	Average Recovery ^d^ (Mean mg/kg ± SD)
**6.25**	5.82 ± 0.28	4.82	11.16	−6.88	93.18 ± 4,49
**12.5**	13.16 ± 0.53	4.09	5.37	5.28	105.28 ± 4.31
**25**	26.05 ± 0.98	3.78	6.35	4.20	104.21 ± 3.94
**50**	48.66 ± 1.89	3.90	7.29	−2.68	97.33 ± 3.80
**100**	103.70 ± 2.27	2.19	5.80	3.70	103.70 ± 2.27

^a^ n = 6 days with three replicates per day. ^b^ Expressed as RSD: RSD (%) = (SD/mean) × 100. ^c^ Trueness % = (observed concentration − nominal concentration)/nominal concentration × 100. ^d^ Recovery % = (observed concentration/nominal concentration) × 100.

**Table 3:** Histamine level determination in fresh fish and fishery products using HPLC-UV and TLC/densitometry methods.

Sample (n)	Histamine Levels (mg/kg)	
	HPLC-UV Method	TLC Method
**Fresh mackerel (4)**	nd 10.74 15.36 20.41	nd nd 11.90 17.89
**Fresh Sardina pilchardus (2)**	9.82 16.32	nd 14.53
**Fresh anchovy (2)**	nd 35.21	nd 39.23
**Fresh tuna (2)**	10.12 15.73	nd 13.25
**Marinated anchovy (2)**	nd 14.23	nd 16.23
**Dry salted anchovy (2)**	17.53 nd	22.36 nd
**Canned mackerel (3)**	13.28 nd nd	12.77 nd nd
**Canned tuna (1)**	nd	nd

n: number of samples analyzed; nd: not detected (LOQ HPLC-UV = 8 mg/kg; LOQ TLC = 10.5 mg/kg).

## References

[1] Burger J., Gochfeld M. (2009). Perceptions of the risks and benefits of fish consumption: Individual choices to reduce risk and increase health benefits. Environ. Res..

[2] Prester L. (2011). Biogenic amines in fish, fish products and shellfish: A review. Food Addit. Contam. Part A.

[3] Maldonado M., Maeyama K. (2013). Simultaneous electrochemical measurement method of histamine and Nτ-methylhistamine by high-performance liquid chromatography–amperometry with o-phthalaldehyde–sodium sulfite derivatization. Anal. Biochem..

[4] Nei D., Nakamura N., Ishihara K., Kimura M., Satomi M. (2017). A rapid screening of histamine concentration in fish fillet by direct analysis in real time mass spectrometry (DART-MS). Food Control.

[5] Tortorella V., Masciari P., Pezzi M., Mola A., Tiburzi S.P., Zinzi M.C., Scozzafava A., Verre M. (2014). Histamine poisoning from ingestion of fish or scombroid syndrome. Case Rep. Emerg. Med..

[6] Colombo F.M., Cattaneo P., Confalonieri E., Bernardi C. (2018). Histamine food poisonings: A systematic review and meta-analysis. Crit. Rev. Food Sci. Nutr..

[7] Kung H.F., Wang T.Y., Huang Y.R., Lin C.S., Wu W.S., Lin C.M., Tsai Y.H. (2009). Isolation and identification of histamine-forming bacteria in tuna sandwiches. Food Control.

[8] Bajc Z., Gačnik K. (2009). Densitometric TLC analysis of histamine in fish and fishery products. JPC J. Planar Chromatogr..

[9] Kounnoun A., Maadoudi M.E., Cacciola F., Mondello L., Bougtaib H., Alahlah N., Amajoud N., EL Baaboua A., Louajri A. (2020). Development and Validation of a High-Performance Liquid Chromatography Method for the Determination of Histamine in Fish Samples Using Fluorescence Detection with Pre-column Derivatization. Chromatographia.

[10] Tahmouzi S., Khaksar R., Ghasemlou M. (2011). Development and validation of an HPLC-FLD method for rapid determination of histamine in skipjack tuna fish (*Katsuwonus pelamis*). Food Chem..

[11] EC (2005). Commission Regulation (EC) No 2073/2005 of 15 Novembre 2005 on the microbiological criteria for foodstuffs. Off. J. Eur. Comm..

[12] EU, European Union (2014). Report EUR 26605 EN. Equivalence Testing of Histamine Methods—Final Report.

[13] Peng J.F., Fang K.T., Xie D.H., Ding B., Yin J.Y., Cui X.M., Zhang Y., Liu J.F. (2008). Development of an automated on-line pre-column derivatization procedure for sensitive determination of histamine in food with high-performance liquid chromatography–fluorescence detection. J. Chromatogr. A.

[14] Hwang B.-S., Wang J.-T., Choong Y.-M. (2003). A rapid gas chromatographic method for the determination of histamine in fish and fish products. Food Chem..

[15] Önal A. (2007). A review: Current analytical methods for the determination of biogenic amines in foods. Food Chem..

[16] Šimat V., Dalgaard P. (2011). Use of small diameter column particles to enhance HPLC determination of histamine and other biogenic amines in seafood. LWT Food Sci. Technol..

[17] Patange S.B., Mukundan M.K., Kumar K.A. (2005). A simple and rapid method for colorimetric determination of histamine in fish flesh. Food Control.

[18] Shakila R.J., Vasundhara T.S., Kumudavally K.V. (2001). A comparison of the TLC-densitometry and HPLC method for the determination of biogenic amines in fish and fishery products. Food Chem..

[19] Sherma J. (2000). Thin-layer chromatography in food and agricultural analysis. J. Chromatogr. A.

[20] AOAC (2005). Association of Official Analytical Chemists. Histamine in Seafood, Fluorimetric Method 977.13. AOAC Off Method.

[21] Tao Z., Sato M., Han Y., Tan Z., Yamaguchi T., Nakano T. (2011). A simple and rapid method for histamine analysis in fish and fishery products by TLC determination. Food Control.

[22] Xie Z., Wang Y., Chen Y., Xu X., Jin Z., Ding Y., Yang N., Wu F. (2017). Tuneable surface enhanced Raman spectroscopy hyphenated to chemically derivatized thin-layer chromatography plates for screening histamine in fish. Food Chem..

[23] Yu H., Zhuang D., Hu X., Zhang S., He Z., Zeng M., Fang X., Chen J., Chen X. (2018). Rapid determination of histamine in fish by thin-layer chromatography-image analysis method using diazotized visualization reagent prepared with p-nitroaniline. Anal. Meth..

[24] Romano A., Klebanowski H., La Guerche S., Beneduce L., Spano G., Murat M.L., Lucas P. (2012). Determination of biogenic amines in wine by thin-layer chromatography/densitometry. Food Chem..

[25] Dang A., Pesek J.J., Matyska M.T. (2013). The use of aqueous normal phase chromatography as an analytical tool for food analysis: Determination of histamine as a model system. Food Chem..

[26] Kaźmierczak D., Ciesielski W., Zakrzewski R. (2006). Application of the Iodine-Azide Procedure for Detection of Biogenic Amines in TLC. J. Liq. Chrom. Relat. Technol..

[27] Arulkumar A., Karthik G., Paramasivam S., Rabie M.A. (2017). Histamine levels in Indian fish via enzymatic, TLC and HPLC methods during storage. J. Food Meas. Charact..

[28] Lapa-Guimaraes J., Pickova J. (2004). New solvent systems for thin-layer chromatographic determination of nine biogenic amines in fish and squid. J. Chromatogr. A.

[29] Shalaby A.R. (1999). Simple, rapid and valid thin layer chromatographic method for determining biogenic amines in foods. Food Chem..

[30] Mantoanelli J.O.F., Gonçalves L.M., Pereira E.A. (2020). Dansyl Chloride as a Derivatizing Agent for the Analysis of Biogenic Amines by CZE-UV. Chromatographia.

[31] Duflos G., Dervin C., Malle P., Bouquelet S. (1999). Relevance of matrix effect in determination of biogenic amines in plaice (*Pleuronectes platessa*) and whiting (*Merlangus merlangus*). J. AOAC Int..

[32] Duflos G., Inglebert G., Himber C., Degremont S., Lombard B., Brisabois A. (2019). Validation of standard method EN ISO 19343 for the detection and quantification of histamine in fish and fishery products using high-performance liquid chromatography. Int. J. Food Microbiol..

[33] Altieri I., Semeraro A., Scalise F., Calderari I., Stacchini P. (2016). European official control of food: Determination of histamine in fish products by a HPLC–UV-DAD method. Food Chem..

[34] Latorre-Moratalla M.L., Bover-Cid S., Veciana-Nogués T., Vidal-Carou M.C. (2009). Thin-layer chromatography for the identification and semi-quantification of biogenic amines produced by bacteria. J. Chromatogr. A.

[35] Directive EC Council (2002). 657/EC of 12 August, 1990 on implementing Council Directive 96/23/EC concerning the performance of analytical methods and the interpretation of results. J. Eur. Communit..

[36] Afnor French Standardization Association (2010). Analysis of Agri-Foodstuffs—Protocol of Characterization for the Validation of a Quantitative Method of Analysis by Construction of an Accuracy Profile.

[37] ISO International Organization for Standardization (2017). Microbiology of the Food Chain-Detection and Quantification of Histamine in Fish and Fishery Products.

